# Primary cervical hydatid cyst: a rare occurrence

**DOI:** 10.1186/1746-1596-7-157

**Published:** 2012-11-17

**Authors:** Nuzhat Sultana, Tariq Khan Hashim, Shahida Yahya Jan, Zahidullah khan, Tahir Malik, Walayat Shah

**Affiliations:** 1 Department of Histopathology, Institute of Basic Medical Sciences, Khyber Medical University, Peshawar, Khyber Pakhtunkhwa, 25000, Pakistan; 2 Department of Pathology, Northwest General Hospital and Research Center, Peshawar, Khyber Pakhtunkhwa, 25000, Pakistan; 3 Department of Neurosurgery, Northwest General Hospital and Research Center, Peshawar, Khyber Pakhtunkhwa, 25000, Pakistan; 4 Department of Radiology, Northwest General Hospital and Research Center, Peshawar, Khyber Pakhtunkhwa, 25000, Pakistan

**Keywords:** Hydatid disease, Cervical hydatid cyst, Occipitocervical mass

## Abstract

Hydatid disease, a parasitic infection is caused by *Echinococcus granulosus*. It has serious impact on health and economy especially in countries where it is endemic. It occurs frequently in liver and lung. The disease is chronic and cyst can localize in different organs. A hydatid cyst occurrence in the head and neck is extremely rare. To know the distribution of disease can help in its control and prevention. We report a case of primary cervical hydatid cyst in 20 year old female. A high index of suspicion is required to diagnose hydatid cyst in rare locations like this. Hydatid cyst should be considered in differential diagnosis of benign swellings of head and neck region, so that it can be managed during surgery to prevent acute anaphylaxis.

**Virtual slides:**

The virtual slides’ for this article can be found here: http://www.diagnosticpathology.diagnomx.eu/vs/4915595218376646

## Background

Hydatid disease is caused by the cestode parasite (tapeworm) *Ecchinococcus granulosus*[1]. It is found frequently in rural areas where domestic livestock-raising is common causing serious health problems [2]. Life cycle of *Taenia echinococcus* requires two distinct mammalian hosts. Dogs, wolves and foxes are the definitive host. Intermediate hosts are herbivores like sheep, cattle, horses and rarely human [3]. Humans are occasionally accidently infected by oral ingestion of tapeworm eggs with contaminated food or water or direct contact with host [4]. The eggs hatch after digestion of the outer capsule in the intestinal mucosa and the larvae penetrate the mucosa, reaching the liver through the portal vein. Most of these embryos become lodged in the hepatic sinusoids, where they either die or develop into one or several hydatid cysts. A systemic spread will occur if the larva passes through the capillary sieve. Hydatid cyst, therefore develops most frequently in the liver (65%), the lungs (25%), out of remaining 10% occurs in muscle, spleen, bones, kidneys, brain, eye, heart, and pancreas [5-8]. Involvement of hydatid cyst is extremely rare in head and neck region even in geographical areas where ecchinococcal infestation is frequent. Only a few cases of hydatid cyst located in neck have been reported in literature [9,10]. Therefore in this case, we wish to draw attention to possibility of the hydatid disease in the neck.

### Case presentation

**Figure 1:**
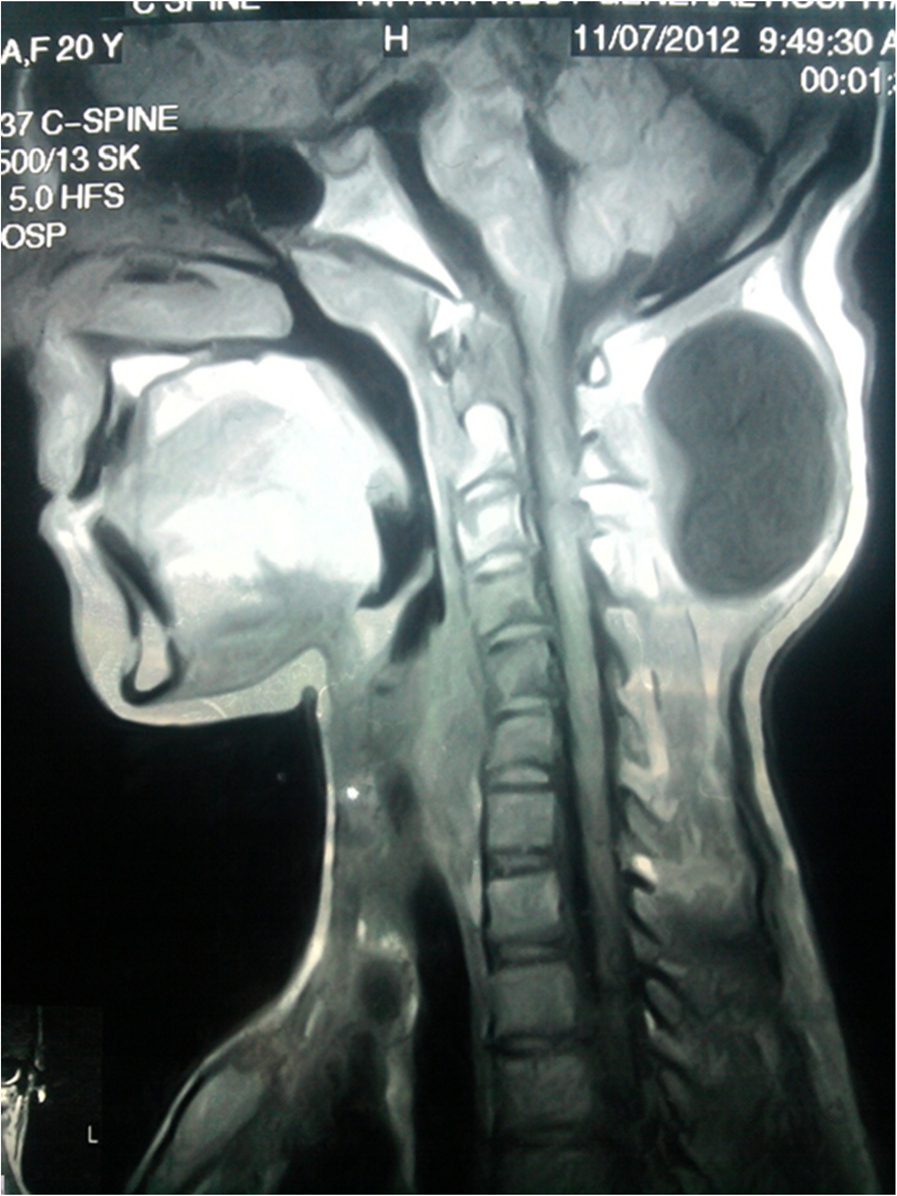
T1 weighted MRI without gadoliniumcontrast shows a hypo-intense cystic mass (6.5x6.1 cm) in the posterior soft tissue at the craniovertebral junction.

**Figure 2:**
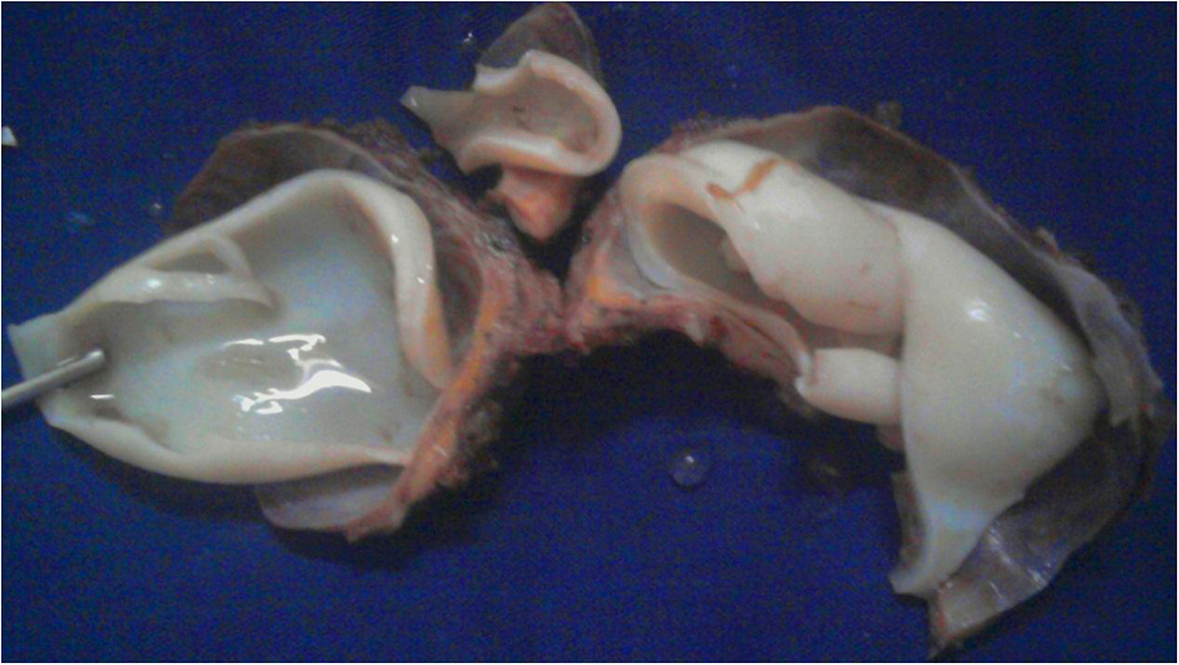
White gelatinous membranous unilocular cyst.

**Figure 3:**
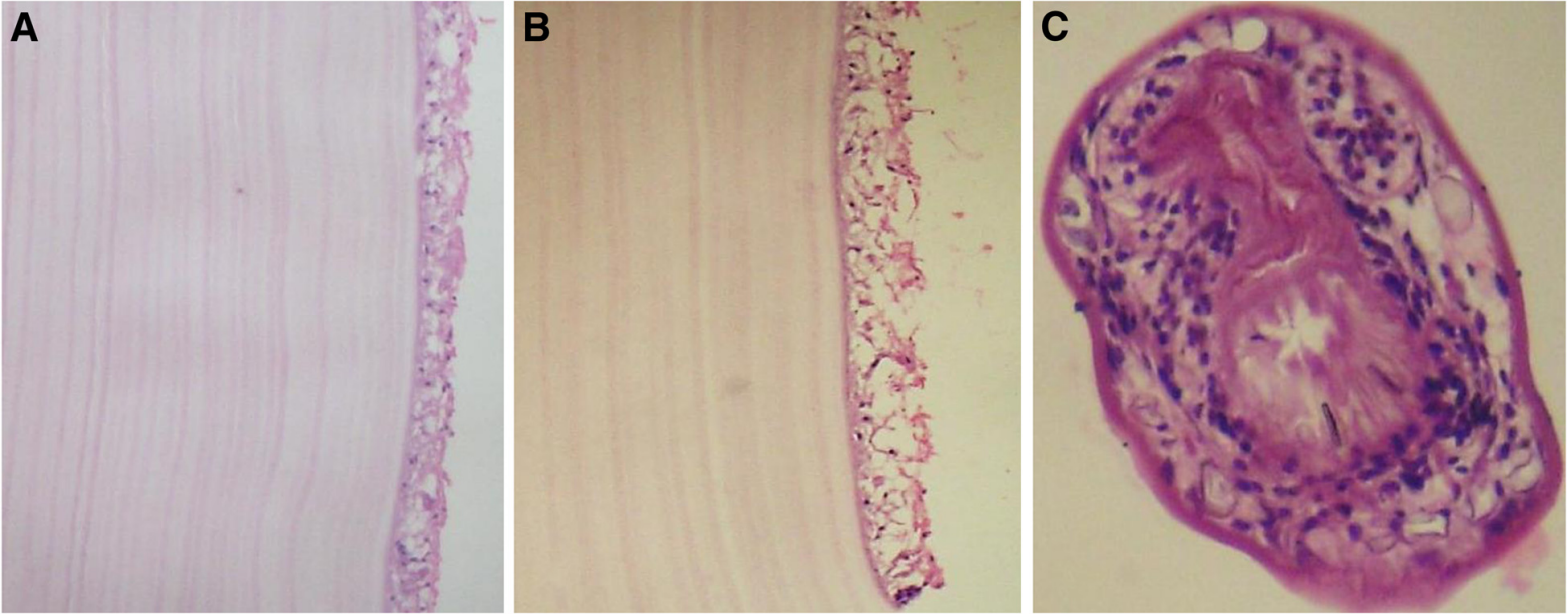
**A-B. Low and High power showing Inner germinal and lamellated layers of cyst wall.C**. Brood capsule.

**Figure 4:**
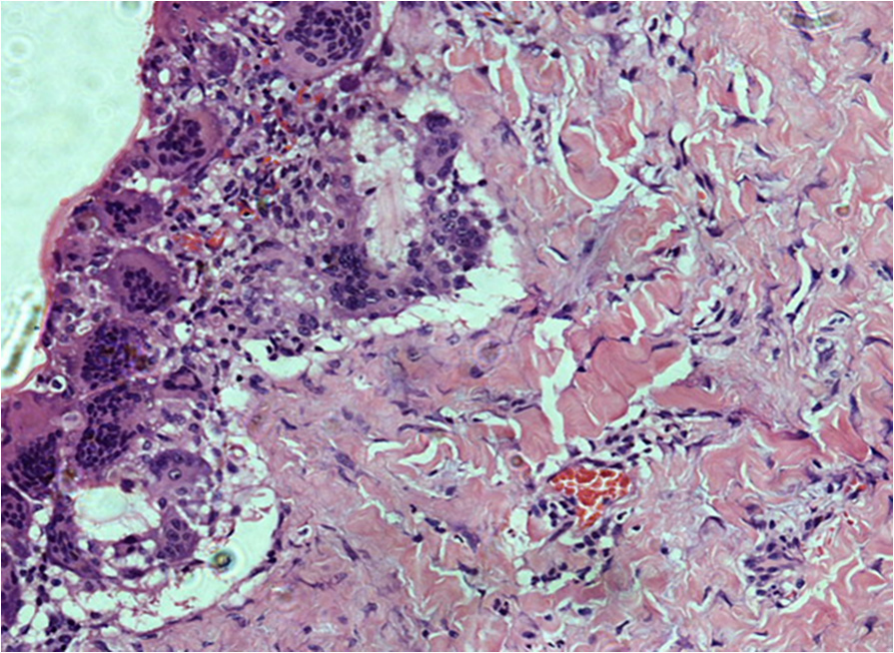
Cyst wall showing foreign body type giant cell reaction.

A 20-year-old female came to neurosurgery department of northwest general hospital with one year history of a slowly growing occipitocervical mass that was painless and without fever. Clinical examination revealed 7x7x4cm round swelling to be cystic and fluctuant with no local inflammatory response or spasm of the cervical muscles. Physical and neurological examinations revealed no abnormalities. Magnetic resonance imaging (MRI) showed a cyst in the muscular plane of upper posterior cervical region, exhibiting low signal on T1W1 with high signal on T2W1 measuring 6.5x6.1 x3.1 cm in size (volume 64 ml) (Figure 1). It has a signal void wall on both T1W1 and T2W1. The cystic lesion was round and well delineated by a thin wall that showed no enhancement after injection of contrast. Both the clinical and laboratory examinations were normal. The patient underwent medical therapies including antibiotics, but her complaints were not alleviated. Her past history was unremarkable. The cyst was removed surgically intact through a posterior approach and there was no spillage of cyst fluid into the surrounding structures. Mass was examined histopathologically. Grossly, there was a single cyst measuring 6.5x 6.5x4 cm in diameter. On sectioning, the cyst was unilocular, filled with white gelatinous membrane (Figure 2). Microscopic examination demonstrated a germinal layer, lamellated ectocyst (Figure 3) with fibrous outer layer. Marked foreign body type giant cell reaction was also seen confirming hydatid cyst (Figure 4).

Ultrasonography (US) of the abdomen with computed tomography (CT) scan of the thorax was done, to know status of other visceral organs but it did not reveal any other organ involvement by the disease process. Patient was given medical treatment with albendazole (800 mg/d) for six weeks.

The outcome was good, with no recurrence after 3months.

## Discussion

Hydatid cyst at unusual sites have been reported around the world including the spleen, kidney, heart, bones, muscles and cranium, but soft tissue hydatid disease represents less than 3% of all hydatid disease [11]. Hydatid disease in cervical region is rare, with only a few cases reported in the literature [9]. At this site, an isolated infestation with no history of cystic rupture elsewhere in the circulation, as seen in our case, is particularly unusual. Hydatid cysts are usually not considered in the differential diagnosis of head and neck cystic swellings, especially in non-endemic areas in the absence of hydatid disease elsewhere in the body. The rarity of the disease in this anatomical location presents a diagnostic difficulty for the physician if he or she is not familiar with the disease.

Hydatid disease may affect humans especially in endemic areas where sheep, dogs and man live in close contact such as Mediterranean countries, the Middle East, South America, New Zealand, Australia, and Southeast Asia and many parts of china [9,12,13]. Unavailability of clean potable water supplies and close association of people with sheep and dogs makes Pakistan a region endemic to the disease [12]. Therefore, a detailed history regarding patient’s occupation, patient’s residence and family history may include diagnosis of hydatid disease in the differential diagnosis. In contrast to the common belief, the incidence of hydatid disease has not decreased significantly during the past decade.

Patients having echinococcosis in any part of the body must undergo thorough systemic investigation as most of the larvae become adult cysts in liver and lung. Multiorgan involvement is seen in 20% to 30% of the cases with involvement of liver in all cases [14]. Our patient did not have any evidence of hydatid disease elsewhere in the body, neither at the time of presentation nor on postoperative diagnostic imaging. Other authors have also confirmed the solitary occurrence of hydatid cysts [9,15]. Interesting aspect of solitary hydatid cysts in the absence of disease in lung and liver is to explain how these larvae produces solitary cysts after passing through the two filter sites. Although no route other than portal has been proven in humans, systemic dissemination through lymphatic route is a strong possibility in case of unusual presentation sites. The majority of hydatid cysts are asymptomatic. Echinococcus in liver produces similar symptoms seen in liver cancer patients like mass, abdominal pain and jaundice. Gal −3, soluble E and N cadherin can be used as marker for differentiation between the two [16]. The values of Gal-3, soluble E cadherin was not estimation in our case. Clinical sign and symptoms depend on the anatomic localization, size and pressure of the enlarged cyst. Therefore signs and symptoms are variable and never pathognomic of hydatid cyst [7]. Hydatid cysts are usually slow-growing, fluctuant, painless masses. These symptoms are characteristic of any slow growing benign tumour in the body. The diameter of the cyst grows at a highly variable rate and ranges from 1 to 5 cm a year [17]. Typically hydatid cyst consists of a single, unilocular cyst, however multiloculated cyst in the same or multiple organ are seen in 20 to 30% of the cases [18].

In non endemic areas because of clinician’s lack of experience and rarity of the disease, it is at times difficult to diagnose a hydatid cyst located in the head and neck region. The diagnosis of *Echinococcus* infection mainly depends on the clinical history of the patient, diagnostic radiological findings and serologic tests. ELISA, Casoni skin tests, latex agglutination, immunoelectrophoresis and direct hemagglutination are serological methods, used for the diagnosis of hydatid disease. Sensitivity of serology is high (80-100%) for liver cysts, but low for lung (50-56%) and other organs (25-56%). Importance of these tests lies mainly in the follow up of treated patients. An increase in titer indicates recurrence of disease and a decrease in titer indicates resolution [9,14,17]. Imaging modalities like US, CT and MRI remain more sensitive than serodiagnosis. These techniques help to determine the cystic avascular nature of the lesion. Daughter cysts, vesicles and internal septa can also be demonstrated [7,19].

No serological test was done in our case because hydatid cyst was not in the clinical differentials. MRI done was not able to do exact characterization and the report was suggestive of benign cyst. The differential diagnosis therefore includes branchial cleft cyst, bronchogenic, thymic, parathyroid, thoracic duct, and foregut-derived (esophageal duplication) cysts, pseudocysts or benign tumor that is congenital and acquired cystic lesions of the neck [2].

For the evaluation of mass lesions in the cervical region, fine-needle aspiration cytology (FNAC) is beneficial however due to possibility of an anaphylactic reaction, dissemination of disease and recurrence as result of spillage of cyst contents, it is not recommended in the routine evaluation of suspected hydatid cysts [15,20,21]. FNAC was not performed in our case due to possibility of benign cystic disease; the case was subjected to surgical excision.

Surgical removal is the most effective treatment of hydatid cyst [9,20]. Surgeon must be careful to avoid spillage of cyst contents to avoid fatal anaphylaxis, recurrence and multiple hydatidosis [7,21]. If presurgical diagnosis is hydatid cyst, preliminary aspiration and instillation of hypertonic saline (20%), silver nitrate (0.5%), formalin, and other chemicals could be used to prevent seeding of the cyst contents and to inactivate the protoscolices [9,17]. Therapy with nontoxic scolocidal agents or combination chemotherapy with mebendazole is of therapeutic value in the treatment of patients with recurrence or a high risk of contamination [15]. There was complete cyst removal with no rupture and spillage of cyst contents in the present case. Albendazole is suggested to be given post operatively for 1–3 months [22]. We treated the present case with albendazole for six weeks post operatively.

The diagnosis of hydatid cyst is confirmed by histology [23]. Histopathological evaluation shows three layers of hydatid cyst. The inner most germinal layer which is thin and translucent on gross. The embryonic tape worm, scolices, develops from an out pouching of the germinal layer and form hydatid sand, settling into the dependent parts of cyst .The cyst fluid is crystal clear, as it is transudate of serum containing proteins and is therefore antigenic. The middle laminated membrane is white 2mm thick and is easily ruptured. It is selectively permeable to nutrients but not to bacteria. The outer layer or pericyst is a rigid protective layer with a few millimeters thickness, representing response of the host to the parasite [21].

Our case also demonstrates histopathologically, scolices, an acellular, thick, lamellar cyst wall. The surrounding host reaction, which is composed of the inflammatory fibrous tissue, forms a dense pseudocapsule around the cyst confirms the diagnosis.

## Conclusion

The case is a reminder that although rare in head and neck, clinicians has to bear in mind Hydatid cyst in the differential diagnosis of cervical masses especially in countries where echinococcus infestation is endemic. Imaging techniques, though sensitive sometimes cannot pin-point the exact etiology of cystic lesion. During surgical removal of cysts in which no definite etiology has been made preoperatively, great care must be taken to avoid spilling of the cystic contents.

### Consent

Written informed consent was obtained from the patient of this case report and accompanying images. A copy of the written consent is available for review by the Editor –In-Chief of this journal.

## Competing interests

The authors declare that they have no competing interests.

## Authors’ contributions

NS and WS participate by conceiving the idea and write the manuscript.TK and ZK performed surgical removal. TM performs radiological interpretation. NS, WS and SY do the pathological exploration. All authors read and approved the final manuscript.
